# Proteomic Assessment of iTRAQ-Based NaoMaiTong in the Treatment of Ischemic Stroke in Rats

**DOI:** 10.1155/2019/5107198

**Published:** 2019-05-15

**Authors:** Kening Li, Minghua Xian, Chi Chen, Shengwang Liang, Lei Chen, Shumei Wang

**Affiliations:** ^1^Department of Traditional Chinese Medicine, Guangdong Pharmaceutical University, Guangzhou 510006, China; ^2^Key Laboratory of Digital Quality Evaluation of Chinese Materia Medica of State Administration of TCM, Guangzhou 510006, China; ^3^Engineering & Technology Research Center for Chinese Materia Medica Quality of the Universities of Guangdong Province, Guangzhou 510006, China

## Abstract

**Background:**

NaoMaiTong (NMT) is widely used in the treatment of cerebral ischemia but the molecular details of its beneficial effects remain poorly characterized.

**Materials and Methods:**

In this study, we used iTRAQ using 2D LC-MS/MS technology to investigate the cellular mechanisms governing the protective effects of NMT. The transient middle cerebral artery occlusion (MCAO) rat model was established and evaluated. The degree of cerebral ischemia was assessed through scoring for nerve injury symptoms and through the assessment of the areas of cerebral infarction. Brain tissues were subjected to analysis by iTRAQ. High-pH HPLC and RSLC-MS/MS analysis were performed to detect differentially expressed proteins (DEPs) between the treatment groups (Sham, MCAO, and NMT). Bioinformatics were employed for data analysis and DEPs were validated by western blot.

**Results:**

The results showed that NMT offers protection to the neurological damage caused by MCAO and was found to reduce the areas of cerebral infarction. We detected 3216 DEPs via mass spectrometry. Of these proteins, 21 displayed altered expression following NMT intervention. These included DEPs involved in translation, cell cycle regulation, cellular nitrogen metabolism, and stress responses. Pathway analysis revealed seven key DEPs that were enriched in ribosomal synthesis pathways, tight junction formation, and regulation of the actin cytoskeleton. According to protein-protein interaction analysis, RPL17, Tuba, and Rac1 were affected by NMT treatment, which was validated by western blot analysis.

**Discussion:**

We therefore identify new pharmacodynamic mechanisms of NMT for the prevention and treatment of ischemic stroke. These DEPs reveal new targets to prevent ischemic stroke induced neuronal damage.

## 1. Introduction

Cerebral ischemia or stroke is a common central nervous system disorder caused by a decreased blood flow to brain tissue that leads to broad cerebral injury and disability [1]. The number of ischemic stroke patients continues to increase, with approximately 800,000 sufferers each year [2]. Tissue plasminogen activator (t-PA) is the only FDA-approved thrombolytic treatment for ischemic stroke [3]. Unfortunately, t-PA has a restrictive time window and can increase the risk of fatal or disabling intracranial hemorrhage [4]. To date, there is little information on the effective stroke treatments. NaoMaiTong (NMT) consists of four traditional Chinese medicines: Rheum palmatum L. (Dahuang), Panax ginseng C.A.Mey. (Renshen), Pueraria lobata (Willd.) Ohwi. (Gegen), and Ligusticum chuanxiong Hort. (Chuanxiong) [5]. NMT is widely used for the prevention and treatment of cardiovascular and cerebrovascular diseases, particularly for the treatment of ischemia cerebral apoplexy, for which it has been used for several decades [5]. Preclinical and pharmacological experiments have shown that NMT exerts a protective effect against cerebral ischemic injury, producing safe and curative effects during the treatment of ischemic stroke [5]. According to previous studies, the protective effects of NMT are related to its ability to antagonize free radical damage, inhibit apoptosis, inhibit cytokine production, and protect from microvascular basement membrane damage [1]. However, traditional approaches are not sufficiently comprehensive to identify multiple targets relevant to complex stroke pathophysiology. Systematic surveys of ischemic brain injury have not been reported, and further research in this area is urgently required [6].

To address this problem, Isobaric Tags for Relative and Absolute Quantitation (iTRAQ) have been applied to systematic investigations, which have a diverse range of applications to effectively capture the quantitative proteome [7]. iTRAQ is a unique in vitro isotopic labeling strategy to simultaneously quantify proteins in up to 8 samples [8]. Moreover, iTRAQ and 2D-LC-MS/MS have been combined, for example, in the proteomic analysis of rats with acute cerebral ischemic stroke using rt-PA [9]. Using these techniques, 18 proteins were found that show significant expressional changes after rt-PA administration, many of which were involved in excitatory neurotransmitter function or cytoskeletal structures. Three key network proteins DPYSL2, RTN4, and NF-kB were identified by the IPA pathway, which is important in the clinical setting of thrombolytic therapy for acute ischemic stroke [9].

In this study, we applied iTRAQ-based proteomics (iTRAQ-2DLC-MS/MS) to label ischemic rat brain tissue samples to identify differentially expressed proteins (DEPs) following NMT treatment. Following this, we probed the function of the DEPs and their influence on related pathways. We provide important insights into the mechanisms of NMT on ischemic stroke and reveal novel candidate therapeutic targets.

## 2. Material and Methods

### 2.1. Materials

#### 2.1.1. Ethics Statement

All experimental procedures were approved by the Animal Ethics Committee of the Guangdong Pharmaceutical University.

### 2.2. Animals

Male Sprague-Dawley (SD) rats weighing 280-300g were purchased from Guangdong Medical Lab Animal Center. All animals were maintained in specific pathogen free grade chambers at 22°C with a relative humidity of 50±5%.

### 2.3. Reagents

iTRAQ reagents, 8plex (4381664), were purchased from AB Sciex. HRP goat anti-rabbit IgG peroxidase-labeled secondary antibodies (SE134) were purchased from Solarbio, anti-*β*-actin antibodies were purchased from Sigma, and anti-RPL17, anti-alpha tubulin, and anti-Rac1 antibodies were purchased from the American Abcam Company (Cambridge, UK). Water was purified using a Millipore Milli‐Q purification system (Bedford, MA, USA). All reagents were of analytical grade.

### 2.4. NMT Preparation

Rhubarb, ginseng, puerarin, and chuanxiong were weighed according to well defined ratios based on the clinical experience of Professor Jiansheng Li (Henan University of Traditional Chinese Medicine, Henan, China) for the treatment of ischemia. The herbal mixture was added to flasks and extracted in 60% ethanol (100 g/1000 mL) at 90°C for 1 hour. This procedure was repeated on two occasions. Extracts were combined, filtered, and concentrated to 0.3125 g/mL to obtain the NMT solution [1].

### 2.5. Rat Model of Cerebral Ischemia Reperfusion (MCAO)

Longao [10] modified method was established to establish MCAO cerebral ischemia reperfusion rat model: rats were anesthetized by intraperitoneal injection of 10% chloral hydrate (300 mg/kg). After complete anesthesia, the rats were fixed in the supine position on the operating table. In the median longitudinal incision in the neck (about 25 mm), the right common carotid artery (CCA) and external carotid artery (ECA) were bluntly isolated, and the proximal end of the CCA and ECA was ligated and the internal carotid artery (ICA) clamped with a micro-arterial clip. Use the ophthalmology scissors to cut the small end of the CCA (about 5 mm from the bifurcation of the common carotid artery) ligation into a small opening and insert the suture, loosen the ICA artery clip, and quickly adjust the strength and direction of the suture. The suture enters the ICA from the CCA until the initial area of the anterior cerebral artery. After the resistance, it is inserted with a little force and then stopped, and the length is about 20~22 mm. Fasten the suture with a suture to fix it, and it was confirmed that the skin was sutured without hemorrhage. After 2 h of ischemia, the suture was slowly withdrawn to the marked black spot to form reperfusion, and the perfusion was continued for 6 h. The sham operation group did not insert the suture line, and the remaining steps were the same as the MCAO model group.

### 2.6. Proteomics Experimental Design

A total of 42 SPF healthy male rats (weight 250-300g) were divided into three groups: sham operation group (n=14), NMT group (n=14), and rat cerebral ischemia reperfusion (MACO) model group (n=14). Rats were dosed with 1 mL/100g for five consecutive days according to body weight. The sham operation and MACO model groups were intragastrically administered physiological saline solution on a daily basis. The NMT group was administered a gavage of NMT at a dose of 3.125g/kg·d. Rats underwent surgical procedures after 1h of final administration (fasting for 12 h). All rats were euthanized through IP euthanyl injection [11].

### 2.7. Tissue Extraction

Frozen cerebra were quickly removed and sectioned into 5 mm slices over ice after reperfusion for 6 h [10]. Brain sections were removed and the remaining brain tissue was frozen in liquid nitrogen and stored at -80°C prior to proteomic analysis. The anterior and posterior of the brain slice were incubated in Tetrazolium Chloride (2% TTC) at 37°C for 20 mins [10]. Images of the infarct were taken and infarct volume was measured using an image analysis system. Neurological impairment was scored according to the Longa 5-point scale.

### 2.8. Proteomics Sample Preparation

From the above experimental results, some brain tissues were screened as proteomic analysis samples, that is, the sham operation group (n=6), the NMT group (n=6), and the MCAO model group (n=6) [1]. The mortar was precooled using liquid nitrogen, and brain tissue was rapidly ground into powder and dissolved into 800 uL of L3 SDS lysis buffer supplemented with PMSF [12]. Samples were sonicated for 5 mins (80 W), and centrifuged for 20 mins (4°C, 12000 r/min) to break up the nucleic acid in the sample. Supernatants were collected into two tubes and 1 mL of acetone was added overnight for protein extraction (-20°C). The next day, samples were centrifuged for 20 min (4°C, 12000 r/min), supernatants were discarded, and the pellet was air-dried at room temperature. Samples were stored at -80°C until use. Prior to assessment, 200-250 *μ*L of L3 buffer was added repeat the above ultrasonic and centrifugal operation, and protein content was assessed via BCA kit [13]. Subsequently, 6 samples of each group were randomly matched and mixed, and two mixed samples from each group were subjected to 3 repeated assessments. Except for the sham operation group, the MCAO model group, the NMT group, and the fourth group which were the internal standard (internal standard of a mixture of all samples), 3 mixed sample proteins were processed as follows: A mixture of 200 *μ*g of protein solution and 4 *μ*L of reducing reagent (iTRAQ reagent) were incubated at 60°C for 1 h and 2 *μ*L of cysteine-blocking reagent was added at room temperature for 10 min. Samples were centrifuged for 20 min (12000 r/min) and 100 *μ*L of 1M TEAB was added to the pellets. Samples were centrifuged for 20 mins. Supernatants were discarded and the process was repeated 3 times. Trypsin was added at a ratio of 1: 100 to 1: 50 for 2 h, and 1M TEAB was added to achieve a final sample volume of 50 *μ*L. Samples were incubated at 37 C for 18 h. Proteins were isolated, 50 *μ*L of 1M TEAB was readded, and samples were centrifuged for 20 min. The two steps were combined to obtain the final sample.

### 2.9. iTRAQ Labeling and 2D LC-MS/MS Analysis

The iTRAQ labels for each group were as follows: 114-sham operation group, 116-NMT group, 118-MCAO model group, and 121-internal standard [10]. iTRAQ labeled samples were analyzed using a Gemini-NX 3u C18 column (250 x 4.6 mm, 5 *μ*m). All experimental samples were monitored in real time [14]. A total of 20 fractions were collected, each of which used RPLC-MS analysis. Data were collected and processed using Thermo Fisher's Thermo Proteome Discoverer 1.4 (Version 1.4.0.288) software. AB Sciex's Protein Pilot TM Software 4.5 was used to process the raw acquired iTRAQ data files. The expression quantity, variation coefficient (CV), and p-values of each protein were analyzed on the basis of the screening criteria for DEPs, classed as a 1.2-fold change in expression (a multiple of the difference in expression of the same protein in both samples), CV ≤ 0.5, p-value < 0.05 for each protein.

### 2.10. Bioinformatics Analysis

DEPs were assessed by Simca 13.0 for principal component analysis (PCA) to verify the screening conditions [12]. The GOs were analyzed from three aspects: describing the molecular function of the gene, the cell region in which the gene was located, and the biological process involved. Pathway enrichment analysis was performed on the DEPs involved pathway using Mas 3.0. String was used for PPI analysis of differential expressed proteins (http://www.string-db.org/, Version 9.1) [7].

### 2.11. Confirmation of DEPs by Western Blot

To confirm the iTRAQ proteomics results, specific DEPs were selected for western blot verification analysis. Protein concentrations were measured with a BCA kit. The total protein was separated with 10% SDS-PAGE. Proteins were electro-transferred to polyvinylidene fluoride (PVDF) membranes. Membranes were blocked with 5% nonfat dry milk in TBST and probed with antibodies against RPL17 (1:1000, Abcam, US), alpha tubulin (1:1000, Abcam, US), Rac1 (1:1000, Abcam, US0), and *β*-Actin antibodies (1:1000, Abcam, US) at 4°C overnight. Membranes were washed and labeled with HRP conjugated goat anti-rabbit IgG secondary antibodies (1:5000, Solarbio, China) at room temperature for 60 mins. Protein bands were visualized with ECL substrate and exposure to X-ray film. Western blotting bands were quantified by Image J software [15].

## 3. Results

### 3.1. Establishment of the MCAO Surgery Model

In total, 42 rats were tested in this experiment and underwent MCAO or sham surgery and tissue extraction [10]. Nine rats died during the experiment and were excluded from analysis. Three rats were excluded due to the infarct size upon TTC staining. For experimental consistency, three samples from the sham operation group were discarded. The remaining 27 rats underwent proteomic analysis, 18 of which were analyzed by iTRAQ-2D LC-MS/MS with 6 rats in each experimental group. Data from 9 rats were verified by western blot analysis (3 rats per experimental group, Table 1).

### 3.2. Nerve Injury Symptom Scores and Cerebral Infarct Size

**Figure 1:**
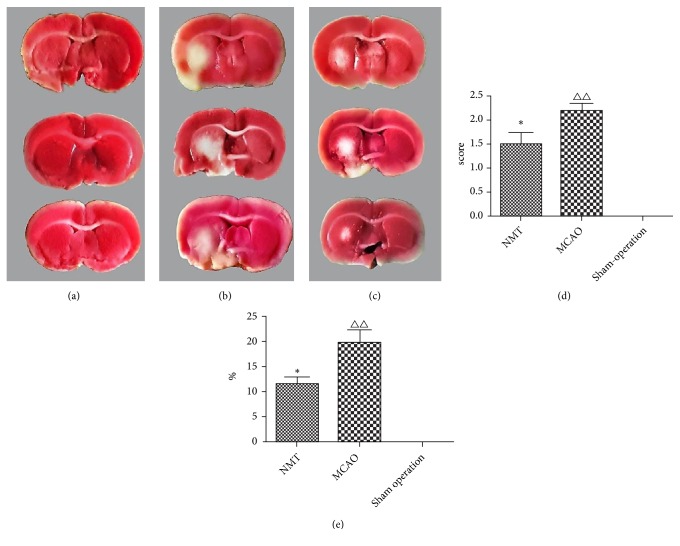
Nerve injury symptom score and cerebral infarct size. (a) Sham operation rats; (b) MCAO rats; (c) MCAO+NMT rats. Red areas reflect living viable tissue. White areas reflect infarcted tissue or areas of white matter. (d) Nerve injury symptom scores. (e) Cerebral infarct size. *∗* is p<0.05 vs. MCAO model group; △△ is p<0.01 vs. sham operation group.

Neurological symptom scores (Figure 1(d)) showed that the NMT group had significant improvement in neurological impairment symptoms compared with the MCAO model group (p<0.05, n=6), indicating that NMT can reduce the symptoms of nerve damage caused by cerebral ischemia. The results of cerebral infarction areas (Figure 1(e)) showed no cerebral infarction in the sham operation group, whilst a marked infarct area was observed in the MCAO model and NMT groups. However, the infarct area of the NMT group was significantly lower than the MCAO group (p<0.05, n=6). This indicated that NMT can reduce the range of cerebral infarction caused by ischemia and hypoxia.

### 3.3. Mass Spectrometry Analysis

**Figure 2:**
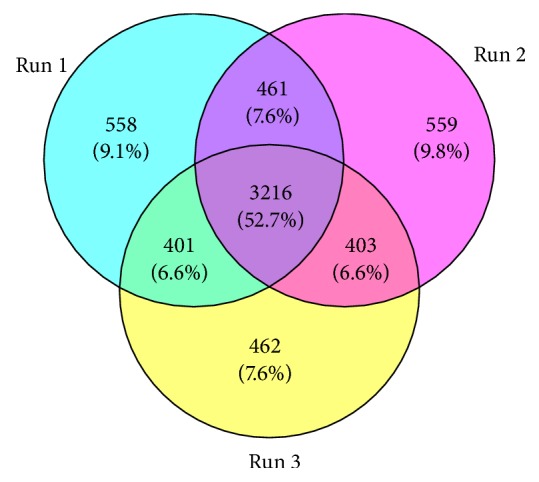
Venn diagram of three duplications.

Through statistical analysis, we used the optimal protein score Unused (ProtScore) as the screening standard. We selected proteins with Unused>1.3 and 95% confidence levels as the analysis object (Table 2). We employed iTRAQ to detect DEPs in NMT treated rats with ischemic stroke. Through searching the Proteinpilot 4.5 database, 3216 proteins (95% protein confidence) were detected across three replicate experiments (Figure 2).

### 3.4. DEP Screening

**Figure 3:**
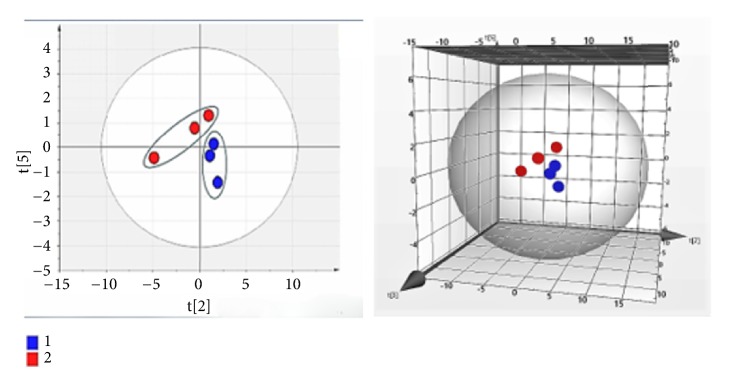
Scatter plot and score scatter 3D plots following PCA analysis. Red represents DEPs of the MCAO model group and blue represents DEPs of the NMT group.

We identified 286 DEPs between the MCAO model and NMT group (Table 3) and 162 DEPs between the MCAO model group and sham operation group. To investigate key proteins related to cerebral ischemia and NMT intervention, we pooled common DEPs between MCAO and sham groups, and NMT and MCAO groups, identifying 21 proteins (Table 4). PCA analysis (Figure 3) of the proteins was performed using Simca 13.0 software, from the score scatter plots and score scatter 3D plots, which indicated that there are significant differences in the expression levels of the DEPs between groups (R^2^X=1, Q^2^=0.988).

### 3.5. Function Annotation and Classification of Differential Proteins by GO

**Figure 4:**
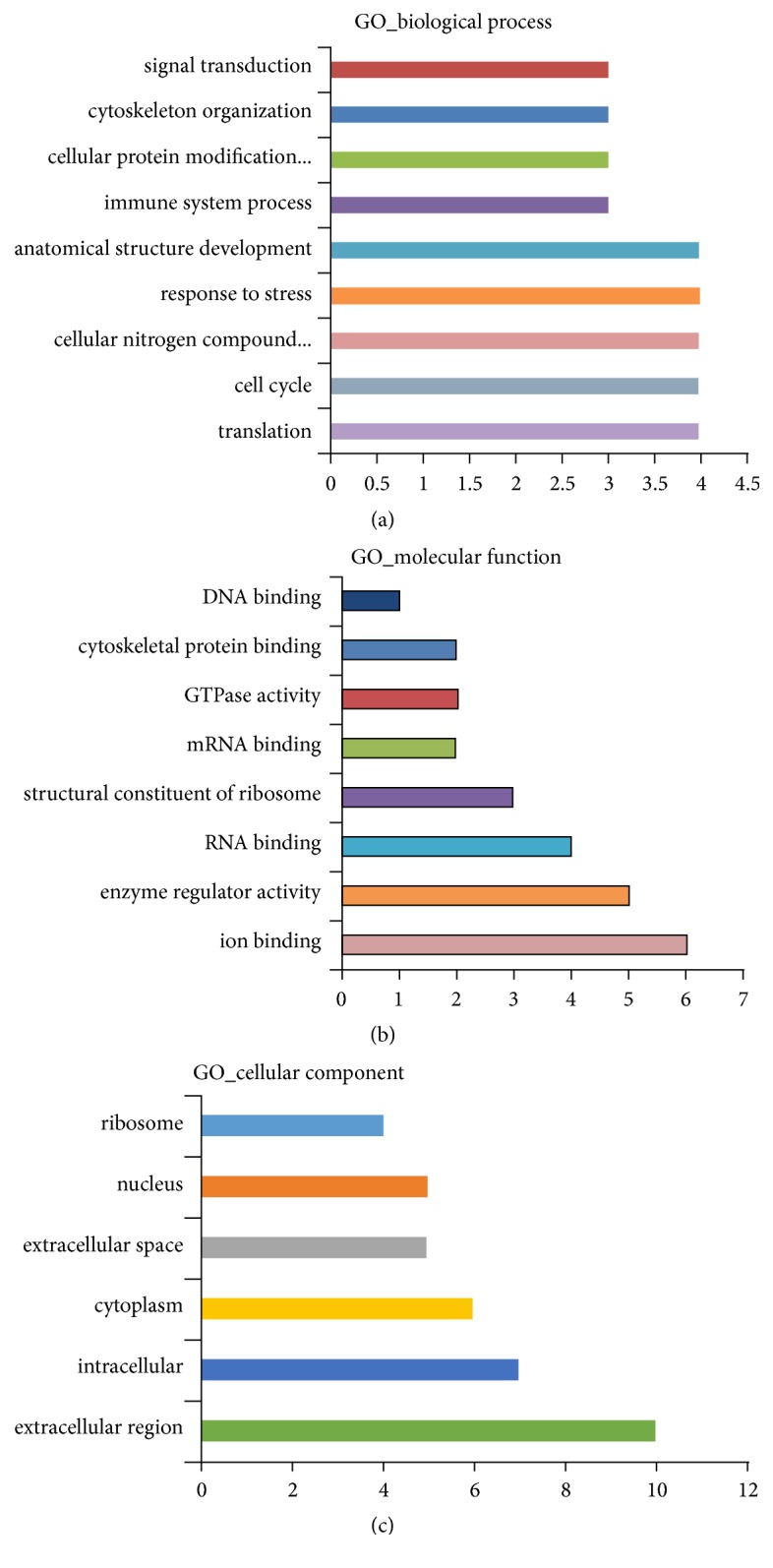
GO analysis. (a) Biological processes; (b) molecular functions; (c) cellular components.

Many cellular proteins have multiple biological functions [16]. To define the biological processes altered following NMT treatment [14], DEP datasets were analyzed using GO protein function annotation and classification (Figure 4). Cell localization analysis revealed that the differentially proteins were mainly intracellular, extracellular, and/or localized to the nucleus. GO cluster analysis identified 21 DEPs participating in protein translation, cell cycle regulation, cellular nitrogen formation, and stress responses (top four). The molecular processes influenced by the 21 DEPs included ion binding, enzyme activity regulation, RNA binding, and ribosome structure (top four).

### 3.6. Pathway Analysis of DEPs

From the pathway analysis, based on the significance of the p-value and the number of enrichment factors, seven key proteins RPL26, RPL17, RPL39, RPS13, Tuba, NWASP, and Rac1 enriched in three important signaling pathways (Table 5) were identified. These included the ribosome pathway, tight junction regulation, and actin cytoskeleton rearrangements.

### 3.7. Protein-Protein Interactions Analysis of DEPs

**Figure 5:**
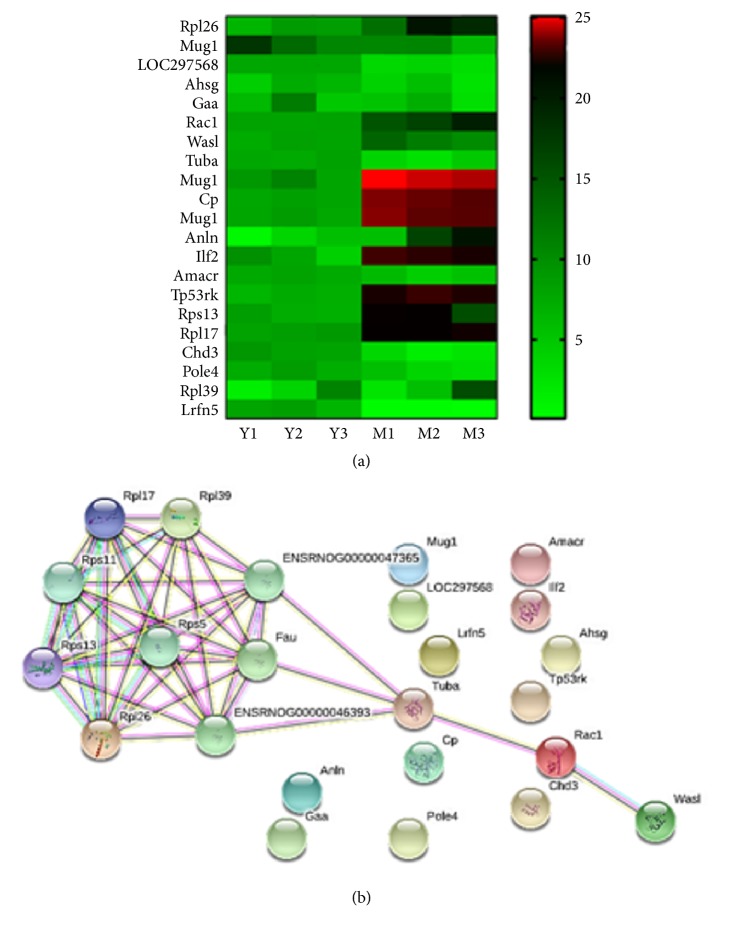
(a) Hierarchical clustering of the quantitative information from 21 DEPs between the MCAO and NMT groups; Y1/2/3: NMT group of 3 duplications; M1/2/3: MCAO group 3 duplication; (b) functional networks of the DEPs.

The 21 DEPs were further analyzed by hierarchical clustering [17]. Clear differences were evident between the NMT group and the MCAO group (Figure 5(a)). Protein-protein interaction (PPI) networks associated with the 21 DEPs were generated using the STRING database (Figure 5(b)). We can see the proteins of Rpl26, Wasl, Rps13, Rpl17, Rpl39, Tuba3a, and Rac1 were tightly networked and other proteins are independent and unrelated. Except for NWASP, the proteins are involved in the above three pathways [18].

### 3.8. Verification by Western Blot

**Figure 6:**
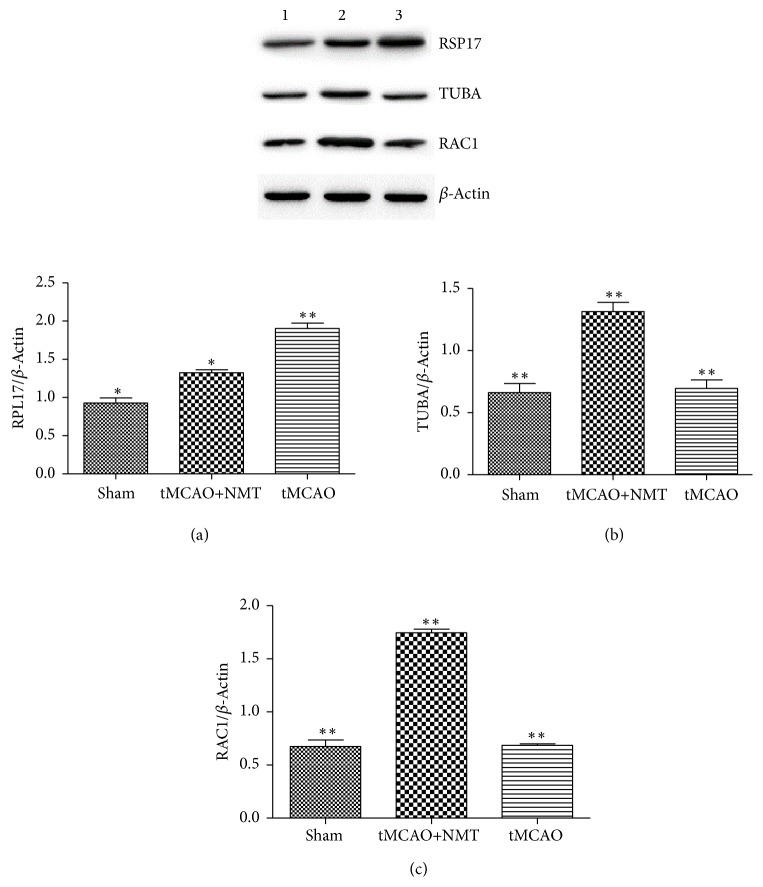
DEPs validation by western blot analysis. (1) Sham operation; (2) MCAO + NMT; (3) MCAO. Band intensity measures are shown for (a) RPL17; (b) TUBA; (c) RAC1. *∗* p <0.05; *∗∗* p < 0.01.

Based on hierarchical clustering analysis, it was found that the two proteins RPL17 and Rac1 were significantly different in the NMT group compared with the MCAO group. By reviewing the literature, we also found that RPL17, Tuba, and Rac1 have a great relationship with the pathogenesis of cerebral ischemia and were each in one of the three more important pathways enriched by 21 DEPs. Therefore, we selected RPL17, Tuba, and Rac1 as significant DEPs with significant differential expression in three pathways for validation by western blot (Figure 6, p<0.05, n=3). The results showed that the MCAO+NMT significantly decreased RPL17 expression and increased Tuba and Rac1 expression compared to MCAO models. This was consistent with the above quantitative proteomic datasets. This validated the identification of the 21 DEPs and confirmed a role RPL17, Tuba, and Rac1 involved in three important pathways in cerebral ischemia.

## 4. Discussion

As we all know, few studies exposed the mechanism of NMT on ischemic stroke. In this study, we used iTRAQ labeling and 2D LC-MS/MS to identify DEPs and altered signaling pathways following ischemic stroke in MCAO rat models [19]. We first confirmed that NMT reduced neurological damage and infarct area compared to the MCAO model group (p< 0.05, n=6) [20]. Using iTRAQ-based proteomic analysis to identify DEPs in the brain tissues of rats receiving NMT, we identified total of 3216 proteins, of which 21 were differentially expressed following NMT administration. GO analysis revealed that the 21 DEPs were localized intracellularly, extracellularly, and/or in the nucleus. The identified DEPs regulated protein translation, cell cycle progression, cellular nitrogen formation, stress responses, enzyme activity, RNA binding, and ribosome structure, amongst other cellular functions. Pathway analysis revealed that seven key DEPs were enriched into three signaling pathways, namely, ribosome function, tight junction regulation, and the actin regulatory network. These included RPL26, RPL17, RPL39, RPS13, Tuba, NWASP, and Rac1, respectively. Western blot data confirmed the differential expression of RPL17, Tuba, and Rac1, which was consistent with the proteomic datasets, confirming their role in NMT-mediated protection during cerebral ischemic stroke.

In this study, we identified three key signaling pathways linked to NMT administration. First of all, four proteins were involved in ribosome function during ischemic stroke, namely, RPL26, RPL17, RPL39, and RPS13. Abnormalities in ribosome synthesis trigger ribosomal stress responses whilst dysfunction of ribosome associated proteins can lead to ribosomal synthesis disorders. The culmination of these effects is to increase the number of free ribosomal proteins, leading to p53 activation [21, 22]. P53 is a proapoptotic gene that is targeted by an array of apoptotic mediators including AIP1, PERP, PIGs, Fas, Apaf-1, Bax, PUMA, and Noxa [23–25]. Apoptosis is intrinsically linked to stroke lesions, which are characterized by a core of cell death that forms rapidly after injury. Cells in the infarct core die within minutes of a stroke whilst in the surrounding region (ischemic penumbra) cell death proceeds more slowly (hours to days). The major types of cell death in the penumbra are apoptosis and autophagy, thereby affecting the process of cerebral ischemia [26, 27]. It was reported that RPL17 alters ribosome maturation leading to cell cycle arrest and aberrant gene transcription [28]. Whilst a role for RPL17 during ischemic has not been reported, our datasets identify this DEP as a new therapeutic target during ischemic stroke therapy.

A second important pathway identified was the tight junction pathway. This involves three distinct proteins: alpha tubulin (Tuba), NWASP, and Rac1 [29]. It has been shown that hypoxia and ischemia reperfusion cause damage to the blood-brain barrier caused by altered expression and localization of tight junction proteins [30]. According to our bioinformatics analysis and western blot data, Tuba was identified as a key DEP related to the NMT-mediated protection of cerebral ischemic stroke. Tuba is a scaffold protein concentrated at neuronal synapses that forms complexes with dynamin and actin regulatory proteins. Tuba is a known substrate of calpain, the activation of which following stroke induced Ca^2+^ influx leads to Tuba depolymerization and cytoskeleton damage. Alteration of the neuronal cell cytoskeleton influences neuronal signal transduction, a major pathological feature of neurodegeneration and neuronal cell death.

Rac1 is an important regulator of the actin cytoskeleton that prevents neuronal apoptosis through promoting cell adhesion, migration, enhancing cell-to-cell signaling, and proliferation. Rac1 is also a biological regulator of NADPH oxidase [22] and has been shown to regulate apoptosis following cerebral ischemia through regulating ROS the production [31, 32]. In this study, Rac1 expression was enhanced in the NMT group compared to the MCAO model group, highlighting this DEP as a potential target for ischemic stroke treatment. Since Rac1 is a multifunction protein with roles in VEGF, MAPK, and Wnt signaling, further studies are required to fully define the cellular functions that contribute to its NMT-mediated protective effects. After reviewing the literature and analyzing the Rac1 protein played a multiple function in these pathways and was a multifunctional protein [6, 33].

In summary, we have identified several candidate cellular proteins/pathways related to the protective effects of NMT during ischemic stroke. Our data suggest that the therapeutic mechanism of NMT is related to its ability to regulate ribosome function following stroke induced downregulation of RPL26, RPL17, RPL39, and RPS13 [32] and through the upregulation of Tuba and Rac1 that activate NWASP to regulate actin function and tight junction formation. These findings reveal new insights into the neuroprotective effects of NMT in acute MCAO rats at the molecular level. Further studies are now required to investigate the cellular roles of the DEPs in response to NMT treatment, as a reference for the future clinical NMT treatment.

## Figures and Tables

**Table 1:** Results of MCAO surgery.

Results/category	Sham operation group	NMT group	MCAO group
Death	2	3	4
TTC staining results are Unqualified	0	2	1
Discard sample	3	0	0
Proteomic analysis	6	6	6
Western blot	3	3	3
Total	14	14	14

**Table 2:** Statistical results of trusted proteins.

Corresponding experimental batch	Number of trusted proteins identified (excluding duplicate proteins)	Number of trusted proteins identified (repeated proteins are not excluded)
Run 1	4636	4667
Run 2	4679	4716
Run 3	4482	4521

**Table 3:** Screening results of DEPs (1.2-fold differential protein).

Group	Up-protein	Down-protein	Total differential protein
MCAO VS NMT	252	34	286
MCAO VS sham operation group	122	40	162

**Table 4:** Information regarding 21 DEPs.

Uniprot ID	Gene name	Protein name	%Cov	Fold Change
G3V6I9	Rpl26	60S ribosomal protein L26	46.6	0.4894 ↓
F1LSG0	Wasl	Neural Wiskott-Aldrich syndrome protein	26.3	0.6486 ↓
A0A0G2K532	Lrfn5	Leucine-rich repeat and fibronectin type-III domain-containing protein 5	28.0	0.1318 ↓
D4AA52	Mug1	Alpha-1-inhibitor III	66.4	0.0394 ↓
G3V7K3	Cp	Ceruloplasmin	31.8	0.0817 ↓
D4A6E3	Mug1	Alpha-1-inhibitor III	47.9	0.0780 ↓
M0RDG0	Anln	Anillin, actin-binding protein	50.1	0.3311 ↓
A0A0H2UHX8	Ilf2	Interleukin enhancer-binding factor 2	39.2	0.1343 ↓
D3ZCD7	Tp53rk	RCG32142, isoform CRA- b	29.3	0.1419 ↓
P62278	Rps13	40S ribosomal protein S13	48.6	0.4740 ↓
F1LZX7	Rpl17	60S ribosomal protein L17	27.7	0.2680 ↓
A0A0H2UI11	Rpl39	60S ribosomal protein L39	46.9	0.7061 **↓**
Q68FR8	Tuba	Tubulin alpha-3 chain	26.6	2.9376 ↑
P70473	Amacr	Alpha-methylacyl-CoA racemase	46.4	1.9409 ↑
F1LPP8	Chd3	Chromodomain helicase DNA-binding protein 3	33.0	3.7325 ↑
B2RYN4	Pole4	DNA polymerase epsilon 4, accessory subunit	16.4	2.0701 ↑
Q03626	Mug1	Murinoglobulin-1	32.5	1.5276 ↑
A0A0G2K926	LOC297568	Alpha-1-inhibitor III	62.5	1.5417 ↑
P24090	Ahsg	Alpha-2-HS-glyco protein	51.7	1.7061 ↑
M0R544	Gaa	Glucosidase, alpha, acid, isoform CRA- a	58.5	1.9953 ↑
A0A0G2K0X4	Rac1	Ras-related C3 botulinum toxin substrate 1	32.5	4.2073 ↑

Fold change is the ratio of the expression of NMT group and MCAO model group.

**Table 5:** Enriched pathways of DEPs.

Pathway	Count	p-Value	q-Value	Gene
Ribosome	4	1.72E-08	3.44E-08	RPl26; RPS13; RPl17; RPl39
Tight junction	3	3.36E-04	2.24E-04	Tuba; Rac1; NWASP
Regulation of actin cytoskeleton	2	2.67E-03	8.90E-04	Rac1; NWASP

## Data Availability

The data used to support the findings of this study are available from the corresponding author upon request.

## Abbreviations

NMT: NaoMaiTong
iTRAQ: Isobaric Tags for Relative and Absolute Quantitation
DEPs: Differentially expressed proteins
t-PA: Tissue plasminogen activator
PPI: Protein-protein interaction.

## Supplementary Materials

Supplementary Materials Early analysis of components of NMT was shown in Figure S1; it was suggested that NMT contained various components and these components were controllable. The data of hierarchical clustering of the quantitative information from 21 DEPs between the MCAO and NMT groups were shown in Table S1. The data of Go analysis of GO-biological process for 21 DEPs were shown in Table S2. The data of Go analysis of GO-cellular component for 21 DEPs were shown in Table S3. The data of Go analysis of GO-molecular function for 21 DEPs were shown in Table S4. The data of pathway analysis of DEPs were shown in Table S5.
