# Supplementary material for: Proteomic Assessment of iTRAQ-Based NaoMaiTong in the Treatment of Ischemic Stroke in Rats
Source: Evid Based Complement Alternat Med. 2019 May 15;2019:5107198. doi: 10.1155/2019/5107198 (PMC6541990; doi:10.1155/2019/5107198)
Supplement: Supplementary Materials — Early analysis of components of NMT was shown in Figure S1; it was suggested that NMT contained various components and these components were controllable. The data of hierarchical clustering of the quantitative information from 21 DEPs between the MCAO and NMT groups were shown in Table S1. The data of Go analysis of GO-biological process for 21 DEPs were shown in Table S2. The data of Go analysis of GO-cellular component for 21 DEPs were shown in Table S3. The data of Go analysis of GO-molecular function for 21 DEPs were shown in Table S4. The data of pathway analysis of DEPs were shown in Table S5. [file 5107198.f1.docx]

Fig. S1 UPLC-MS/MS TIC map of NMT extract liquid

(positive ion mode and negative ion mode)

Table S1 The data of Hierarchical clustering of 21 DEPs

| Gene | Y1 | Y2 | Y3 | M1 | M2 | M3 |
| --- | --- | --- | --- | --- | --- | --- |
| Rpl26 | 0.8317637 | 1.076465 | 1.047129 | 1.50558 | 2.3956071 | 2.189618 |
| Mug1 | 2.0892961 | 1.570363 | 1.282331 | 1.2769842 | 1.2993614 | 0.794416 |
| LOC297568 | 0.963829 | 0.9727472 | 0.963829 | 0.4851728 | 0.5309575 | 0.4251728 |
| Ahsg | 0.5861382 | 0.9120108 | 0.8165824 | 0.5435544 | 0.7345588 | 0.3786252 |
| Gaa | 0.7870458 | 1.380384 | 0.6367955 | 0.6944498 | 0.8918178 | 0.4191478 |
| Rac1 | 1 | 0.990832 | 1.009253 | 1.7712202 | 1.953148 | 2.2894599 |
| Wasl | 0.9120108 | 1.018591 | 0.990832 | 1.6061221 | 1.3704456 | 1.2276472 |
| Tuba3a | 0.963829 | 0.937562 | 1.009253 | 0.5281008 | 0.3991592 | 0.635638 |
| Mug1 | 1.106624 | 1.318257 | 0.9817479 | 25.086904 | 20.458299 | 17.91746 |
| Cp | 0.963829 | 1.028016 | 0.9817479 | 13.797172 | 11.582815 | 10.016498 |
| Mug1 | 0.9727472 | 1.066596 | 0.9549926 | 14.471118 | 10.674308 | 10.243495 |
| Anln | 0.1753881 | 0.510505 | 0.7311391 | 0.7297134 | 1.9418454 | 2.4082124 |
| Ilf2 | 1.191242 | 0.9727472 | 0.5754399 | 8.0700073 | 6.2430916 | 4.647347 |
| Amacr | 0.9462371 | 1 | 0.9289664 | 0.775249 | 0.5852249 | 0.7086266 |
| Tp53rk | 0.8165824 | 0.9289664 | 0.887156 | 4.7546327 | 7.5466272 | 5.2519803 |
| Rps13 | 1.047129 | 0.9036494 | 0.8709636 | 3.209133 | 2.9064333 | 1.8374759 |
| Rpl17 | 1 | 1.047129 | 1.086426 | 3.0313433 | 2.9071979 | 3.9538284 |
| Chd3 | 1.116863 | 1.009253 | 0.990832 | 0.4992265 | 0.270396 | 0.3654607 |
| Pole4 | 0.9120108 | 1.066596 | 0.8953648 | 0.7405636 | 0.5152389 | 0.4325225 |
| Rpl39 | 0.258226 | 0.5248075 | 1.3061709 | 0.3657074 | 0.7432481 | 1.8498385 |
| Lrfn5 | 0.9817479 | 1.037528 | 0.8317637 | 0.1293944 | 0.1367462 | 0.1096265 |

Table S2 The date of GO-biological process

| GO-ID | GO-Name | Protein-Accessions | Protein-id-num | Aspect |
| --- | --- | --- | --- | --- |
| GO:0022607 | cellular component assembly | A0A0G2JZB8, D3ZTB4, F1LMW7, Q9JJ19, Q9R0I8 | 5 | Biological-process |
| GO:0007165 | signal transduction | Q1JU68, Q9JJ19, M0R3V4, P27274 | 4 | Biological-process |
| GO:0008219 | cell death | D4A5L9, Q9JJ19, M0R3V4, P27274 | 4 | Biological-process |
| GO:0008283 | cell proliferation | Q9JJ19, M0R3V4, Q5FVI4, P27274 | 4 | Biological-process |
| GO:0030154 | cell differentiation | Q4V898, Q9JJ19, Q5FVI4, Q9R0I8 | 4 | Biological-process |
| GO:0034641 | cellular nitrogen compound metabolic process | D4A5L9, Q4V7C6, Q4V898, M0R3V4 | 4 | Biological-process |
| GO:0048856 | anatomical structure development | A0A0G2JZB8, Q9JJ19, Q5FVI4, Q9R0I8 | 4 | Biological-process |
| GO:0002376 | immune system process | B4F7A5, Q9R0I8, P27274 | 3 | Biological-process |
| GO:0006412 | translation | Q1JU68, D3ZEI0, P62268 | 3 | Biological-process |
| GO:0006461 | protein complex assembly | D3ZTB4, F1LMW7, Q4V898 | 3 | Biological-process |
| GO:0006464 | cellular protein modification process | Q1JU68, Q9JJ19, M0R3V4 | 3 | Biological-process |
| GO:0006810 | transport | D3ZTB4, Q9JJ19, G3V6R0 | 3 | Biological-process |
| GO:0007010 | cytoskeleton organization | A0A0G2JZB8, F1LMW7, Q9JJ19 | 3 | Biological-process |
| GO:0009058 | biosynthetic process | Q4V7C6, Q4V898, M0R3V4 | 3 | Biological-process |
| GO:0022618 | ribonucleoprotein complex assembly | Q1JU68, Q4V898, P62268 | 3 | Biological-process |
| GO:0055085 | transmembrane transport | Q9JJ19, G3V6R0, P35171 | 3 | Biological-process |
| GO:0006091 | generation of precursor metabolites and energy | D4A5L9, P35171 | 2 | Biological-process |
| GO:0006605 | protein targeting | D3ZTB4, Q9JJ19 | 2 | Biological-process |
| GO:0007155 | cell adhesion | A0A0G2JZB8, P27274 | 2 | Biological-process |
| GO:0007267 | cell-cell signaling | D3ZPJ0, Q9JJ19 | 2 | Biological-process |
| GO:0044281 | small molecule metabolic process | D4A5L9, Q4V7C6 | 2 | Biological-process |
| GO:0048646 | anatomical structure formation involved in morphogenesis | M0R3V4, Q5FVI4 | 2 | Biological-process |
| GO:0048870 | cell motility | Q9JJ19, Q5FVI4 | 2 | Biological-process |
| GO:0000902 | cell morphogenesis | Q9JJ19 | 1 | Biological-process |
| GO:0006397 | mRNA processing | Q4V898 | 1 | Biological-process |
| GO:0006520 | cellular amino acid metabolic process | Q4V7C6 | 1 | Biological-process |
| GO:0006629 | lipid metabolic process | Q9R0I8 | 1 | Biological-process |
| GO:0006913 | nucleocytoplasmic transport | Q9JJ19 | 1 | Biological-process |
| GO:0006914 | autophagy | Q9R0I8 | 1 | Biological-process |
| GO:0006950 | response to stress | P27274 | 1 | Biological-process |
| GO:0007005 | mitochondrion organization | D3ZTB4 | 1 | Biological-process |
| GO:0007009 | plasma membrane organization | Q9JJ19 | 1 | Biological-process |
| GO:0007034 | vacuolar transport | D3ZTB4 | 1 | Biological-process |
| GO:0007049 | cell cycle | Q9JJ19 | 1 | Biological-process |
| GO:0009056 | catabolic process | D3ZTB4 | 1 | Biological-process |
| GO:0009790 | embryo development | Q9JJ19 | 1 | Biological-process |
| GO:0016192 | vesicle-mediated transport | D3ZTB4 | 1 | Biological-process |
| GO:0021700 | developmental maturation | Q5FVI4 | 1 | Biological-process |
| GO:0030198 | extracellular matrix organization | A0A0G2JZB8 | 1 | Biological-process |
| GO:0034330 | cell junction organization | A0A0G2JZB8 | 1 | Biological-process |
| GO:0042592 | homeostatic process | Q9JJ19 | 1 | Biological-process |
| GO:0044403 | symbiosis, encompassing mutualism through parasitism | Q1JU68 | 1 | Biological-process |
| GO:0050877 | neurological system process | Q9JJ19 | 1 | Biological-process |
| GO:0051604 | protein maturation | P27274 | 1 | Biological-process |
| GO:0061024 | membrane organization | D3ZTB4 | 1 | Biological-process |

Table S3 The date of GO-cellular component

| GO-ID | GO-Name | Protein-Accessions | Protein-id-num | Aspect |
| --- | --- | --- | --- | --- |
| GO:0005576 | extracellular region | F1LMW7, Q4V898, Q9JJ19, M0R3V4, P35171, P27274 | 6 | Cellular-component |
| GO:0005737 | cytoplasm | D3ZTB4, F1LMW7, Q1JU68, Q4V7C6, Q9JJ19, Q9R0I8 | 6 | Cellular-component |
| GO:0005829 | cytosol | D4A5L9, Q1JU68, Q4V7C6, D3ZEI0, P62268 | 5 | Cellular-component |
| GO:0005615 | extracellular space | F1LM05, Q4V898, M0R3V4, P27274 | 4 | Cellular-component |
| GO:0005634 | nucleus | F1LMW7, Q1JU68, Q4V898, Q9R0I8 | 4 | Cellular-component |
| GO:0005886 | plasma membrane | D3ZPJ0, Q9JJ19, Q9R0I8, P27274 | 4 | Cellular-component |
| GO:0005622 | intracellular | Q4V898, D3ZEI0, P62268 | 3 | Cellular-component |
| GO:0005739 | mitochondrion | D4A5L9, P35171, Q6AXY8 | 3 | Cellular-component |
| GO:0005856 | cytoskeleton | D3ZTB4, F1LMW7, Q1JU68 | 3 | Cellular-component |
| GO:0005840 | ribosome | D3ZEI0, P62268 | 2 | Cellular-component |
| GO:0043234 | protein complex | D3ZTB4, Q1JU68 | 2 | Cellular-component |
| GO:0000228 | nuclear chromosome | Q4V898 | 1 | Cellular-component |
| GO:0005730 | nucleolus | Q1JU68 | 1 | Cellular-component |
| GO:0005764 | lysosome | D3ZTB4 | 1 | Cellular-component |
| GO:0005768 | endosome | D3ZTB4 | 1 | Cellular-component |
| GO:0005773 | vacuole | Q9R0I8 | 1 | Cellular-component |
| GO:0005783 | endoplasmic reticulum | Q6AXY8 | 1 | Cellular-component |
| GO:0005815 | microtubule organizing center | F1LMW7 | 1 | Cellular-component |
| GO:0005929 | cilium | Q9JJ19 | 1 | Cellular-component |
| GO:0031410 | cytoplasmic vesicle | D3ZTB4 | 1 | Cellular-component |

Table S4 The date of GO-molecular function

| GO-ID | GO-Name | Protein-Accessions | Protein-id-num | Aspect |
| --- | --- | --- | --- | --- |
| GO:0003723 | RNA binding | Q1JU68, Q4V898, D3ZUL1, P62268 | 4 | Molecular-function |
| GO:0019899 | enzyme binding | F1LMW7, Q1JU68, Q4V7C6, Q9JJ19 | 4 | Molecular-function |
| GO:0043167 | ion binding | D4A5L9, Q4V7C6, Q9R0I8 | 3 | Molecular-function |
| GO:0003729 | mRNA binding | Q1JU68, Q4V898 | 2 | Molecular-function |
| GO:0003735 | structural constituent of ribosome | D3ZEI0, P62268 | 2 | Molecular-function |
| GO:0008092 | cytoskeletal protein binding | F1LMW7, Q9JJ19 | 2 | Molecular-function |
| GO:0022857 | transmembrane transporter activity | G3V6R0, P35171 | 2 | Molecular-function |
| GO:0003677 | DNA binding | Q4V898 | 1 | Molecular-function |
| GO:0005198 | structural molecule activity | Q9JJ19 | 1 | Molecular-function |
| GO:0008135 | translation factor activity, RNA binding | Q1JU68 | 1 | Molecular-function |
| GO:0016301 | kinase activity | Q9R0I8 | 1 | Molecular-function |
| GO:0016491 | oxidoreductase activity | P35171 | 1 | Molecular-function |
| GO:0016874 | ligase activity | Q4V7C6 | 1 | Molecular-function |
| GO:0030674 | protein binding, bridging | D3ZTB4 | 1 | Molecular-function |

Table S5 The date of KEGG analysis

| Pathway | Count | p-Value | q-Value | Gene | Input Symbol |
| --- | --- | --- | --- | --- | --- |
| Ribosome | 4 | 1.72E-08 | 3.44E-08 | Rpl26; Rps13; Rpl17; Rpl39 | Rpl26; Rps13; Rpl17; Rpl39 |
| Adherens junction | 2 | 0.000336 | 0.000224 | Rac1; Wasl | Rac1; Wasl |
| Regulation of actin cytoskeleton | 2 | 0.00267 | 0.00089 | Rac1; Wasl | Rac1; Wasl |
| Galactose metabolism | 1 | 0.00793 | 0.00176 | Gaa | Gaa |
| Porphyrin and chlorophyll metabolism | 1 | 0.0136 | 0.00216 | Cp | Cp |
| DNA polymerase | 1 | 0.0136 | 0.00216 | Pole4 | Pole4 |
| Starch and sucrose metabolism | 1 | 0.0154 | 0.00216 | Gaa | Gaa |
| Nucleotide excision repair | 1 | 0.0162 | 0.00216 | Pole4 | Pole4 |
| Base excision repair | 1 | 0.0199 | 0.00239 | Pole4 | Pole4 |
| VEGF signaling pathway | 1 | 0.0266 | 0.00239 | Rac1 | Rac1 |
| Renal cell carcinoma | 1 | 0.027 | 0.00239 | Rac1 | Rac1 |
| Amyotrophic lateral sclerosis (ALS) | 1 | 0.027 | 0.00239 | Rac1 | Rac1 |
| Fc epsilon RI signaling pathway | 1 | 0.0273 | 0.00239 | Rac1 | Rac1 |
| Pancreatic cancer | 1 | 0.0273 | 0.00239 | Rac1 | Rac1 |
| B cell receptor signaling pathway | 1 | 0.0299 | 0.00239 | Rac1 | Rac1 |
| Pyrimidine metabolism | 1 | 0.031 | 0.00239 | Pole4 | Pole4 |
| Gap junction | 1 | 0.034 | 0.00239 | Tuba3a | Tuba3a |
| Toll-like receptor signaling pathway | 1 | 0.0347 | 0.00239 | Rac1 | Rac1 |
| Colorectal cancer | 1 | 0.0347 | 0.00239 | Rac1 | Rac1 |
| Leukocyte transendothelial migration | 1 | 0.0431 | 0.00239 | Rac1 | Rac1 |
| Axon guidance | 1 | 0.0467 | 0.00239 | Rac1 | Rac1 |
| Purine metabolism | 1 | 0.0514 | 0.00239 | Pole4 | Pole4 |
| Wnt signaling pathway | 1 | 0.0518 | 0.00239 | Rac1 | Rac1 |
| Natural killer cell mediated cytotoxicity | 1 | 0.0525 | 0.00239 | Rac1 | Rac1 |
| Focal adhesion | 1 | 0.069 | 0.00255 | Rac1 | Rac1 |
| MAPK signaling pathway | 1 | 0.0942 | 0.00304 | Rac1 | Rac1 |
